# Collargolum Enemata

**Published:** 1904-09

**Authors:** 


### ABSTRACTS.

### Collargolum Enemata.

Dr. Heinrich Loebl, Assistant in the Franz Joseph Hospital at Vienna, in *Die Therapie der Gegenwart,* April, 1904, says: that during the last few years there has been a constant increase in the number of favorable collargolum publications. Good results have been recorded from its use in apparently absolutely hopeless cases, and we convinced ourselves, in our division, of the fact that anyone who administers it to a considerable number of septic patients will have the same experience. In serious cases we did not employ unguentum Credé, preferring the intravenous injection of 1 per cent. collargolum solutions (2¼ grains to ½ oz. sterile water), which we used with excellent results.

But great obesity, anemia, venous paucity, and the like, may hinder the intravenous injection. Besides, a single injection is rarely enough, and only a limited number can be given if a new vein is used for each. Finally, the needle may be displaced from the lumen of the vein by a motion of the patient when the constricting bandage is loosened.

In one case where the veins could not be reached on account of their anemic narrowness and the patient's well developed panniculous adiposus, he used collargolum as an enema. The method has the following palpable advantages: it can be used by any attendant, is neither painful nor troublesome, is entirely safe, permits the use of larger doses, and brings the drug in contact with an excellent absorbing surface. It is no less efficacious than the intravenous injection. The intestine is not irritated, even when 1 per cent. solutions are given for fourteen days, and the collargolum enema is almost entirely absorbed, for a cleansing enema given twelve hours later brings only a slightly blackish fluid.

Having given the enema to two dying patients and demonstrated the absorption of collargolum by historical examination, Loebl used the enemata in 27 cases, which are detailed with illustrative temperature charts. Six were sepses of varied origin; of these, 4 were cured, 1 was not benefited, and 1 died. One of the cured cases was a staphylococcemia in which other remedies had failed; and one other case is especially noteworthy because it appeared hopeless when the collargolum was begun. The fatal case was so desperate that the surgeon refused to operate, and even it showed some improvement the next day, so that a less marantic individual (she was 65 years old and tubercular) might have been saved. Out of nine cases of puerperal infection five were cured, three were not benefited, and one died. In this series one beginning parametric infiltration with irritative peritoneal symptoms underwent rapid involution and in five other cases there was an inhibitive effect on suppuration. In one case of obstinate rheumatism and in four cases of renal and vesical infection there were no results. Six consumptives with rapidly progressing febrile phthisis, one of them moribund and all of them entirely recalcitrant to every internal medication, received the enemata without any especial effect. In sanatoria, where there is naturally a larger proportion of mild cases, there is probably a greater chance of success.

As regards the technic, the patient is given morning and evening an enema of 1 or 2 pints of lukewarm water. Half an hour after defecation, 7½ grains of collargolum in 1 per cent. solution are administered by a funnel or syringe. This amount (1½ ozs.) is almost invariably retained; the bowels usually move only after the cleansing clyster on the next morning. The enemata are given twice daily for at least eight and not more than fourteen days. No unpleasant by-effects have been observed. Loebl concludes that the collargolum enemata give the same results in acute infections as does unguentum Credé of collargolum intravenously. In many cases the method has a surprising effect; and it has more than once been effectual when joint affections or phlegmasia alba dolens had appeared. It is probable that the method will save cases which we would otherwise lose.

For these reasons he heartily recommends a trial of the treatment. Since November, 1903, when he demonstrated the method in the Royal and Imperial Medical Society of Vienna, he has received many favorable reports on it, both in hospital and in private practice.

---

Prof. Netter, Member of the Paris Academy of Medicine, writes in the *Bulletins et Mémoires de la Societe des Hopitaux* of April 28, 1904, concerning the administration of collargolum by mouth and per rectum. He finds that the gastrointestinal route, which Loebl and Jousset have used successfully, has the advantage of simplicity and often gives excellent results. By mouth he gives pills of 1-6 to 1-3 grain collargolum with sugar of milk and glycerin; or solutions in albuminised water, ¾ grain to a dessertspoonful, the daily dose being 2½ drams to 1 oz.

For rectal injections 1½ to 7½ grains collargolum should be given twice daily. Or 1½ grain to 4½ grain collargolum suppositories may be used. Several of his patients have taken collargolum per os and per rectum for months at a time, one without interruption for a year.

In noninfectious diseases, such as nervous and gastric affections, in which silver nitrate is used, collargolum acts well, has no caustic effect, and never causes argyria. He gave it to six epileptics and got the specific action of bromide from much smaller than the usual dose; and the distressing symptoms of bromism and the eruptions, bad breath and mental depression were avoided. He even used collargolum alone and patients have passed five or six months without any attack.

He also used it successfully in several cases of neurasthenia and neuralgia. A chorea that was intractable to antipyrin and arsenic was cured by it and it was also effectual in migraine. Given by mouth in infectious intestinal diseases, dysentery, and muco-membranous enteritis of children and adults, it gave marvelously rapid results.

The internal use of collargolum is also preferable to injection or inunction in infectious diseases with a prolonged course, such as phthisis pulmonum. In typhoid especially the drug has a direct effect upon the pathogenic organisms, diminishing fever and diarrhea and shortening the disease. Results in influenza were equally good. In certain tuberculous cases collargolum alone appeared useful. Cases with cavities seemed especially benefited; in several there was rapid diminution of expectoration. In infectious endocarditis subsequent to biliary inflammation the prolonged use of the drug certainly lowered temperature and increased the patient's strength.

